# Determinants for patient satisfaction regarding aesthetic outcome and skin sensitivity after breast-conserving surgery

**DOI:** 10.1186/s12957-016-1053-8

**Published:** 2016-12-07

**Authors:** Cecilia Dahlbäck, Jonas Manjer, Martin Rehn, Anita Ringberg

**Affiliations:** 1 Department of Plastic and Reconstructive Surgery, Skåne University Hospital, Jan Waldenströmsgata 18, 205 02 Malmö, Sweden; 2 Department of Clinical Sciences, Lund University, Malmö, Sweden; 3 Breast Unit, Department of Surgery, Skåne University Hospital, Malmö, Sweden

**Keywords:** Breast cancer, Breast-conserving surgery, Patient satisfaction, Aesthetics, Sensitivity

## Abstract

**Background:**

With the development of new surgical techniques in breast cancer, such as oncoplastic breast surgery, increased knowledge of risk factors for poor satisfaction with conventional breast-conserving surgery (BCS) is needed in order to determine which patients to offer these techniques to. The aim of this study was to investigate patient satisfaction regarding aesthetic result and skin sensitivity in relation to patient, tumour, and treatment factors, in a consecutive sample of patients undergoing conventional BCS.

**Methods:**

Women eligible for BCS were recruited between February 1, 2008 and January 31, 2012 in a prospective setup. In all, 297 women completed a study-specific questionnaire 1 year after conventional BCS and radiotherapy. Potential risk factors for poor satisfaction were investigated using logistic regression analysis.

**Results:**

The great majority of the women, 84%, were satisfied or very satisfied with the overall aesthetic result. The rate of satisfaction regarding symmetry between the breasts was 68% and for skin sensitivity in the operated breast it was 67%. Excision of more than 20% of the preoperative breast volume was associated with poor satisfaction regarding overall aesthetic outcome, as was axillary clearance. A high BMI (≥30 kg/m^2^) seemed to affect satisfaction with symmetry negatively. Factors associated with less satisfied patients regarding skin sensitivity in the operated breast were an excision of ≥20% of preoperative breast volume, a BMI of 25–30 kg/m^2^, axillary clearance, and radiotherapy. Re-excision and postoperative infection were associated with lower rates of satisfaction regarding both overall aesthetic outcome and symmetry, as well as with skin sensitivity.

**Conclusions:**

Several factors affect patient satisfaction after BCS. A major determinant of poor satisfaction in this study was a large excision of breast volume. If the percentage of breast volume excised is estimated to exceed 20%, other techniques, such as oncoplastic breast surgery, with or without contralateral surgery, or mastectomy with reconstruction, may be considered.

**Electronic supplementary material:**

The online version of this article (doi:10.1186/s12957-016-1053-8) contains supplementary material, which is available to authorized users.

## Background

Breast-conserving surgery (BCS), i.e. a partial mastectomy, followed by radiotherapy, is today a common alternative to mastectomy, when treating early breast cancer.

One intention, when choosing BCS, is to optimize the aesthetic result. Surgical techniques have been developed to improve the aesthetic outcome, while maintaining oncological safety, i.e. oncoplastic breast surgery [1, 2]. However, use of these techniques requires specially trained breast surgeons and, with certain procedures, the participation of a plastic surgeon. Hence, it is important to identify factors associated with a poor aesthetic outcome after conventional BCS, in order to determine which patients would benefit the most from oncoplastic breast surgery. Potential risk factors for a poor aesthetic outcome, including tumour and anthropometric characteristics, as well as treatment modalities, have been studied previously [3–14]. However, patient selection, sample size, studied factors, and ways to evaluate outcome vary greatly between studies.

Another intention when choosing BCS is to preserve skin sensitivity in the operated breast. However, little is known about potential determinants for impaired sensitivity in the breast skin or potential means to minimize this disadvantage.

The aims of this prospective study were to examine patient satisfaction concerning aesthetic result, including symmetry, and skin sensitivity in the breast, in a consecutive sample of patients undergoing conventional BCS at a single institution, and to investigate potential risk factors for low satisfaction.

## Methods

### Study cohort

**Fig. 1:**
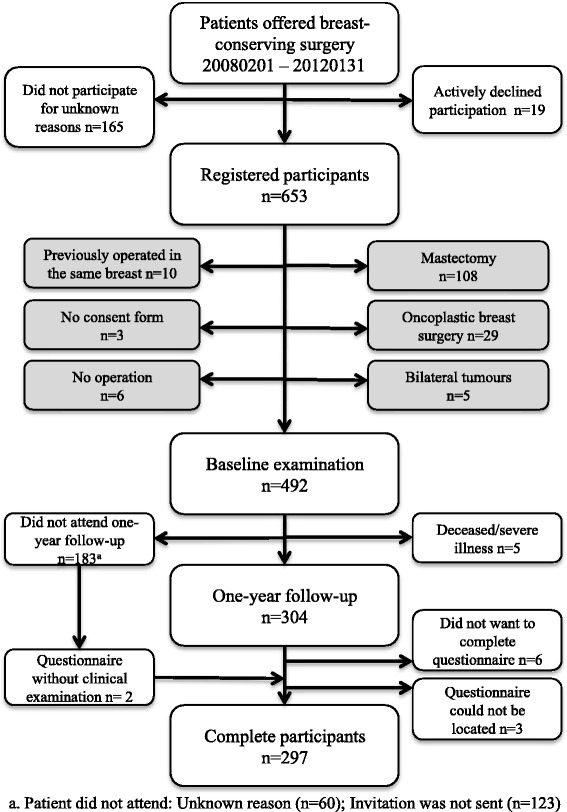
Study cohort

Between February 1, 2008 and January 31, 2012, all patients offered BCS at Skåne University Hospital, Malmö, due to breast cancer or suspected malignancy, and able to comprehend information given in spoken and written Swedish were eligible for inclusion in the present study. A total of 653 patients were identified as potential participants and subsequently registered in the study database (Fig. 1).

Women operated with mastectomy were excluded (*n* = 108). A primary mastectomy was performed in 24 cases on the patient’s request or due to cancer-related reasons, whereas in 84 cases, the mastectomy was performed after BCS due to histopathological findings, such as widespread cancer in situ, multifocality, and/or non-radical margins (*n* = 78), instead of re-excision (*n* = 4), or as a risk-reducing procedure due to high-risk genes (*n* = 2). Other reasons for exclusion were oncoplastic breast surgery (*n* = 29), bilateral tumours (*n* = 5), previous breast cancer surgery in the same breast (*n* = 10), cancelled operation (*n* = 6), or lack of a consent form (*n* = 3). In total, 297 patients completed the questionnaire.

To examine the proportion of identified potential participants in the study compared to all potentially eligible patients, the material was compared retrospectively to the Swedish Breast Cancer Registry. This is managed by the six Regional Cancer Centres in Sweden through the Information Network for Cancer Care (INCA), which reports a very high inclusion rate (98.1%) [15]. It was found that 78% of potential participants had been registered for the current study. Calculation details are presented in the supplemental material (Additional file 1: p. 1-2; Figure S1).

### Baseline examination

The attending surgeon performed the preoperative examination. Height was measured to the closest half centimetre. Weight was measured in kilogrammes to one decimal place. Bilateral breast volume was measured in millilitres, using specially designed and validated plastic cups [16, 17]. Tumour size was measured in millimetres. In cases of non-palpable tumours, the size was established by ultrasound and/or mammography. The location of the tumour was estimated to the closest clock hour, in addition to the “central” position.

### Surgery and adjuvant treatment

The surgeon chose operative method after discussing with the patient. Breast-conserving surgery was generally recommended to women diagnosed with a unifocal breast tumour, less than 4 cm in diameter, if the surgeon considered it possible to achieve an acceptable aesthetic result. Oncoplastic breast surgery techniques were discussed in cases of a large tumour in relation to breast size.

Mobilization of breast tissue from the pectoral fascia and overlying skin was routine. Non-palpable tumours were localized by a hook-wire, placed with ultrasound or stereotactic mammographic guidance before surgery. Six breast surgeons performed 99% of the operations. The sentinel node technique was routinely used in the examination of the axilla. A radioactive isotope (99mTc-Nanocoll) with blue dye was injected near the tumour in all cases. Sentinel nodes (1–3) were localized by a gamma detector and/or the blue dye. They were surgically removed and sent for frozen section analysis. If metastases were found, an axillary clearance was performed.

In the operating theatre, a nurse weighed the excised tissue to the closest gramme. The estimated percentage of breast volume excised (EPBVE) was calculated by comparing the specimen weight to the preoperative breast volume, assuming a one to one correlation between weight and volume. This correlation has been established in previous studies [6, 18, 19] and is considered to be a reasonable approximation for this study.

Chemotherapy, radiotherapy, and hormonal treatment were given according to national guidelines [20]. No patient received neoadjuvant chemotherapy. Adjuvant radiotherapy was administered to the remaining breast parenchyma: 50 Gy per 25 fractions or 42.5 Gy per 16 fractions depending on patient age and tumour characteristics. Women younger than 40 with invasive cancer received a 16 Gy boost to the affected breast quadrant. No patient had brachytherapy. A subgroup of women (*n* = 20) had been enrolled in a parallel ongoing trial, which studied the oncological outcome of BCS without radiotherapy, for women over 65 years of age. Patient charts were reviewed in order to determine which adjuvant treatment had been administered.

### Follow-up examinations and questionnaire

The surgeon examining the patient postoperatively assessed complications. Evacuation of seromas and hematomas was noted. Infection was defined as the presence of clinical symptoms and administered antibiotics, with or without a positive bacterial culture. Only infection was included in the analysis of outcome, due to the lack of reliable data regarding other complications, apart from those leading to surgery.

Patients were invited to follow-up approximately 1 year after the operation and completion of radiotherapy. A trained nurse measured body weight and bilateral breast volume, in the same way as was done preoperatively. The patients were handed a study-specific questionnaire in Swedish (translated version presented in the supplemental material (Additional file 1: p. 3-4)), in which they were asked to rate satisfaction concerning different aspects of the aesthetic outcome (i.e. overall aesthetic outcome, symmetry between the breasts, shape and size of the operated breast, as well as appearance of the scar) and skin sensitivity in the operated breast. The possible ratings were dissatisfied (1), not entirely satisfied (2), satisfied (3), or very satisfied (4). The clinicians who planned the study constructed the questionnaire in consultation with a psychologist. Readability was checked in a pilot test of five women prior to the start of the study.

### Statistical methods

Patient satisfaction was investigated in relation to possible determinants using logistic regression. Outcome was dichotomized into “not satisfied” (1 + 2) or “satisfied” (3 + 4). Odds ratios (OR) were obtained, with 95% confidence intervals (CI). The results were adjusted for age and BMI. A final multivariable model included age and BMI together with factors that were statistically significant in either of the two previous models.

Continuous values were categorized into subgroups. Tumour location, apart from the central position, was grouped into quadrants, considering 12 o’clock to represent the upper outer quadrant.

Statistical analysis was performed using IBM SPSS® Statistics for Macintosh, Version 22.0. Armonk, NY: IBM Corp.

## Results

### Clinical characteristics

Median age at the time of operation was 62 years (interquartile range (IQR) 54–68) and median BMI was 26 kg/m^2^ (IQR 23–29). Median preoperative breast size was 500 ml (IQR 375–737.5), tumour size 15 mm (IQR 10–20), specimen weight 63 g (IQR 45–91), and EPBVE 12.4% (IQR 9.2–17.1) (Additional file 1: Table S1). Infection was recorded in 23 (7.7%) cases. Ten patients required aspiration of seromas, and two hematomas were evacuated. No operations caused by skin necrosis were noted.

### Patient satisfaction

The median time from operation to follow-up was 16 months (IQR 15–18). The proportion of women who were satisfied or very satisfied with the overall aesthetic result was 84%, regarding symmetry between the breasts, it was 68%, and with skin sensitivity in the operated breast it was 67% (Table 1).

**Table 1:** Patient satisfaction

	Very satisfied *n* (%)	Satisfied *n* (%)	Not entirely satisfied *n* (%)	Dissatisfied *n* (%)	Missing *n* (%)
Aesthetic outcome	123 (41.4)	126 (42.4)	28 (9.4)	5 (1.7)	15 (5)
Symmetry	74 (24.9)	128 (43.1)	47 (15.8)	14 (4.7)	34 (11.4)
Skin sensitivity	82 (27.6)	117 (39.4)	65 (21.9)	8 (2.7)	25 (8.4)
Shape of op. breast	98 (33.0)	139 (46.8)	30 (10.1)	4 (1.3)	26 (8.8)
Size of op. breast	94 (31.6)	142 (47.8)	36 (12.1)	2 (0.7)	23 (7.7)
Appearance of scar	122 (41.1)	118 (40.0)	27 (9.0)	6 (2.0)	24 (8.1)

### Aesthetic outcome

An EPBVE ≥20%, axillary clearance, re-excision to obtain clear margins, and postoperative infection increased the risk of poor satisfaction regarding the overall aesthetic result. Axillary clearance remained statistically significant in the multivariable model (Table 2).

**Table 2:** Satisfaction regarding aesthetic outcome

Factor	Satisfied	Not satisfied	OR (95% CI)	OR (95% CI)^a^	OR (95% CI)^b^
	*n* (%)	*n* (%)			
Age (years)					
<50	42 (16.9)	4 (12.1)	1	1	1
≥50–<65	101 (40.6)	18 (54.5)	1.87 (0.60–5.86)	1.84 (0.58–5.78)	2.02 (0.61–6.71)
≥65	106 (42.6)	11 (33.3)	1.09 (0.33–3.61)	0.99 (0.30–3.32)	0.96 (0.27–3.38)
BMI (kg/m^2^)					
<25	111 (44.5)	12 (36.4)	1	1	1
≥25–<30	83 (33.5)	13 (39.4)	1.45 (0.63–3.34)	1.58 (0.68–3.69)	1.57 (0.63–3.91)
≥30	54 (21.8)	8 (24.2)	1.37 (0.53–3.55)	1.49 (0.57–3.92)	1.68 (0.59–4.78)
Missing	1				
Breast volume (ml)^c^					
<500	107 (43.7)	14 (42.4)	1	1	
≥500	138 (56.3)	19 (57.6)	1.05 (0.51–2.20)	1.02 (0.43–2.39)	
Missing	4				
Tumour size (mm)^c^					
<15	120 (48.2)	14 (42.4)	1	1	
≥15	129 (51.8)	19 (57.6)	1.26 (0.61–2.63)	1.16 (0.55–2.45)	
Specimen weight (g)^c^					
<63	127 (51.0)	11 (33.3)	1	1	
≥63	122 (49.0)	22 (66.7)	2.08 (0.97–4.48)	2.15 (0.96–4.83)	
EPBVE^d^ (%)					
<10	78 (31.8)	7 (21.2)	1	1	1
≥10–<20	137 (55.9)	16 (48.5)	1.30 (0.51–3.30)	1.29 (0.50–3.33)	1.14 (0.42–3.07)
≥20	30 (12.2)	10 (30.3)	3.71 (1.30–10.65)	3.93 (1.34–11.55)	3.05 (0.98–9.47)
Missing	4				
Axillary clearance					
No	212 (85.5)	22 (66.7)	1	1	1
Yes	36 (14.5)	11 (33.3)	2.94 (1.32–6.59)	3.02 (1.32–6.90)	2.87 (1.20–6.83)
Missing	1				
Re-excision					
No	234 (94.0)	28 (84.8)	1	1	1
Yes	15 (6.0)	5 (15.2)	2.79 (0.94–8.25)	3.09 (1.01–9.48)	3.02 (0.92–9.89)
Infection					
No	235 (94.4)	28 (84.8)	1	1	1
Yes	14 (5.6)	5 (15.2)	3.00 (1.00–8.95)	2.92 (0.94–9.04)	2.65 (0.83–8.50)
Radiotherapy					
No	37 (14.9)	3 (9.1)	1	1	
Yes	212 (85.1)	30 (90.9)	1.75 (0.51–6.01)	1.48 (0.42–5.26)	
Chemotherapy					
No	220 (88.4)	29 (87.9)	1	1	
Yes	29 (11.6)	4 (12.1)	1.05 (0.34–3.19)	1.10 (0.34–3.54)	
Hormonal therapy					
No	112 (45.0)	13 (39.4)	1	1	
Yes	137 (55.0)	20 (60.6)	1.26 (0.60–2.64)	1.25 (0.59–2.66)	
Quadrant					
UOQ	127 (51.2)	20 (60.6)	1	1	
LOQ	49 (19.8)	4 (12.1)	0.52 (0.17–1.61)	0.51 (0.16–1.57)	
LIQ	20 (8.1)	4 (12.1)	1.30 (0.40–4.26)	1.29 (0.39–4.24)	
UIQ	50 (20.2)	5 (15.2)	0.61 (0.22–1.73)	0.59 (0.21–1.67)	
Central	2 (0.8)	0 (0.0)	–	–	
Missing	1				

^a^Adjusted for age and BMI. ^b^Adjusted for age, BMI, EPBVE, axillary clearance, re-excision, and infection. ^c^Groups divided at the median. ^d^Estimated percentage of breast volume excised

*UOQ* upper outer quadrant, *LOQ* lower outer quadrant, *LIQ* lower inner quadrant, *UIQ* upper inner quadrant

### Symmetry

Obese patients (BMI ≥30 kg/m^2^), those with a specimen weight exceeding 63 g, those who had undergone re-excision, and patients subjected to postoperative infection were less satisfied with symmetry between the breasts. Re-excision remained statistically significant in the multivariable analysis (Table 3).

**Table 3:** Satisfaction regarding symmetry between the breasts

Factor	Satisfied	Not satisfied	OR (95% CI)	OR (95% CI)^a^	OR (95% CI)^b^
	*n* (%)	*n* (%)			
Age (years)					
<50	35 (17.3)	11 (18.0)	1	1	1
≥50–<65	83 (41.1)	26 (42.6)	1.00 (0.44–2.24)	0.97 (0.43–2.19)	0.93 (0.40–2.17)
≥65	84 (41.6)	24 (39.3)	0.91 (0.40–2.05)	0.77 (0.33–1.77)	0.70 (0.30–1.67)
BMI (kg/m^2^)					
<25	95 (47.3)	20 (32.8)	1	1	1
≥25–<30	66 (32.8)	23 (37.7)	1.66 (0.84–3.26)	1.72 (0.87–3.42)	1.68 (0.82–3.44)
≥30	40 (19.9)	18 (29.5)	2.14 (1.02–4.46)	2.26 (1.07–4.80)	1.95 (0.85–4.48)
Missing	1				
Breast volume (ml)^c^					
<500	93 (46.5)	22 (36.1)	1	1	
≥500	107 (53.5)	39 (63.9)	1.54 (0.85–2.79)	1.29 (0.65–2.55)	
Missing	2				
Tumour size (mm)^c^					
<15	97 (48.0)	25 (41.0)	1	1	
≥15	105 (52.0)	36 (59.0)	1.33 (0.75–2.38)	1.29 (0.71–2.34)	
Specimen weight (g)^c^					
<63	111 (55.0)	21 (34.4)	1	1	1
≥63	91 (45.0)	40 (65.6)	2.32 (1.28–4.22)	2.10 (1.12–3.94)	1.74 (0.90–3.34)
EPBVE^d^ (%)					
<10	66 (33.0)	16 (26.2)	1	1	
≥10–<20	109 (54.5)	34 (55.7)	1.29 (0.66–2.51)	1.45 (0.73–2.88)	
≥20	25 (12.5)	11 (18.0)	1.82 (0.74–4.44)	2.11 (0.84–5.29)	
Missing	2				
Axillary clearance					
No	169 (84.1)	49 (80.3)	1	1	
Yes	32 (15.9)	12 (19.7)	1.29 (0.62–2.70)	1.16 (0.55–2.48)	
Missing	1				
Re-excision					
No	192 (95.0)	52 (85.2)	1	1	1
Yes	10 (5.0)	9 (14.8)	3.32 (1.28–8.60)	4.08 (1.53–10.88)	3.30 (1.19–9.14)
Infection					
No	192 (95.0)	53 (86.9)	1	1	1
Yes	10 (5.0)	8 (13.1)	2.90 (1.09–7.71)	2.55 (0.93–6.99)	2.51 (0.91–6.98)
Radiotherapy					
No	28 (13.9)	5 (8.2)	1	1	
Yes	174 (86.1)	56 (91.8)	1.80 (0.66–4.89)	1.82 (0.65–5.10)	
Chemotherapy					
No	178 (88.1)	54 (88.5)	1	1	
Yes	24 (11.9)	7 (11.5)	0.96 (0.39–2.35)	0.87 (0.34–2.23)	
Hormonal therapy					
No	95 (47.0)	21 (34.4)	1	1	
Yes	107 (53.0)	40 (65.6)	1.69 (0.93–3.07)	1.69 (0.92–3.10)	
Quadrant					
UOQ	101 (50.0)	35 (58.3)	1	1	
LOQ	40 (19.8)	10 (16.7)	0.72 (0.33–1.59)	0.67 (0.30–1.51)	
LIQ	17 (8.4)	6 (10.0)	1.02 (0.37–2.79)	1.08 (0.39–3.02)	
UIQ	42 (20.8)	10 (15.0)	0.62 (0.27–1.40)	0.60 (0.26–1.37)	
Central	2 (1.0)	0 (0.0)	–	–	
Missing		1			

^a^Adjusted for age and BMI. ^b^Adjusted for age, BMI, specimen weight, re-excision, and infection. ^c^Groups divided at the median. ^d^Estimated percentage of breast volume excised

*UOQ* upper outer quadrant, *LOQ* lower outer quadrant, *LIQ* lower inner quadrant, *UIQ* upper inner quadrant

### Skin sensitivity

Factors associated with poor patient satisfaction with skin sensitivity in the breast were BMI ≥25–<30 kg/m^2^, tumour size ≥15 mm, EPBVE ≥20%, axillary clearance, re-excision, radiotherapy, and infection. Multivariable analysis showed that BMI ≥25–<30 kg/m^2^, tumour size ≥15 mm, re-excision, and infection were independent risk factors (Table 4).

**Table 4:** Satisfaction regarding skin sensitivity in the operated breast

Factor	Satisfied	Not satisfied	OR (95% CI)	OR (95% CI)^a^	OR (95% CI)^b^
	*n* (%)	*n* (%)			
Age (years)					
<50	33 (16.6)	13 (17.8)	1	1	1
≥50–<65	78 (39.2)	36 (49.3)	1.17 (0.55–2.49)	1.15 (0.54–2.48)	1.15 (0.49–2.71)
≥65	88 (44.2)	24 (32.9)	0.69 (0.32–1.52)	0.61 (0.27–1.36)	0.59 (0.25–1.44)
BMI (kg/m^2^)					
<25	93 (47.0)	26 (35.6)	1	1	1
≥25–<30	62 (31.3)	31 (42.5)	1.79 (0.97–3.30)	1.99 (1.06–3.72)	2.07 (1.04–4.12)
≥30	43 (21.7)	16 (21.9)	1.33 (0.65–2.73)	1.51 (0.72–3.16)	1.78 (0.78–4.03)
Missing	1				
Breast volume (ml)^c^					
<500	88 (44.7)	31 (43.1)	1	1	
≥500	109 (55.3)	41 (56.9)	1.07 (0.62–1.84)	1.05 (0.56–1.98)	
Missing	2	1			
Tumour size (mm)^c^					
<15	106 (53.3)	22 (30.1)	1	1	1
≥15	93 (46.7)	51 (69.9)	2.64 (1.49–4.68)	2.43 (1.36–4.34)	1.88 (1.01–3.48)
Specimen weight (g)^c^					
<63	106 (53.3)	30 (41.1)	1	1	
≥63	93 (46.7)	43 (58.9)	1.63 (0.95–2.81)	1.65 (0.92–2.94)	
EPBVE^d^ (%)					
<10	67 (34.0)	17 (23.6)	1	1	1
≥10–<20	107 (54.3)	40 (55.6)	1.47 (0.77–2.81)	1.49 (0.77–2.90)	1.15 (0.56–2.36)
≥20	23 (11.7)	15 (20.8)	2.57 (1.11–5.96)	2.80 (1.18–6.65)	1.84 (0.73–4.63)
Missing	2	1			
Axillary clearance					
No	171 (85.9)	53 (73.6)	1	1	1
Yes	28 (14.1)	19 (26.4)	2.19 (1.13–4.23)	2.07 (1.05–4.07)	1.57 (0.75–3.28)
Missing		1			
Re-excision					
No	191 (96.0)	63 (86.3)	1	1	1
Yes	8 (4.0)	10 (13.7)	3.80 (1.43–10.02)	4.14 (1.53–11.20)	3.86 (1.34–11.06)
Infection					
No	190 (95.5)	63 (86.3)	1	1	1
Yes	9 (4.5)	10 (13.7)	3.35 (1.30–8.62)	3.58 (1.34–9.60)	3.26 (1.18–9.03)
Radiotherapy					
No	32 (16.1)	5 (5.5)	1	1	1
Yes	167 (83.9)	69 (94.5)	3.31 (1.13–9.70)	2.87 (0.96–8.61)	1.73 (0.53–5.68)
Chemotherapy					
No	180 (90.5)	60 (82.2)	1	1	
Yes	19 (9.5)	13 (17.8)	2.05 (0.96–4.41)	1.94 (0.86–4.36)	
Hormonal therapy					
No	94 (47.2)	26 (35.6)	1	1	
Yes	105 (52.8)	47 (64.4)	1.62 (0.93–2.82)	1.56 (0.89–2.74)	
Quadrant					
UOQ	103 (52.0)	36 (49.3)	1	1	
LOQ	39 (19.7)	14 (19.2)	1.03 (0.50–2.11)	1.03 (0.49–2.15)	
LIQ	16 (8.1)	7 (9.6)	1.25 (0.48–3.29)	1.37 (0.51–3.69)	
UIQ	38 (19.2)	16 (21.9)	1.21 (0.60–2.42)	1.13 (0.56–2.30)	
Central	2 (1.0)	0 (0.0)	–	–	
Missing	1				

^a^Adjusted for age and BMI. ^b^Adjusted for age, BMI, tumour size, EPBVE, axillary clearance, re-excision, and infection. ^c^Groups divided at the median. ^d^Estimated percentage of breast volume excised

*UOQ* upper outer quadrant, *LOQ* lower outer quadrant, *LIQ* lower inner quadrant, *UIQ* upper inner quadrant

### Shape and size of the operated breast and visual appearance of the scar

Women with a BMI ≥25–<30 kg/m^2^ and those with an EPBVE ≥20% were less satisfied with the shape of the operated breast. Axillary clearance was associated with poor satisfaction with the size of the operated breast, but the association did not remain statistically significant after adjustment. A BMI ≥30 kg/m^2^ and an EPBVE ≥20% were associated with less satisfaction regarding the size of the operated breast, but the results were only statistically significant in the adjusted models. Patients with an EPBVE ≥20% were less satisfied with the scar (Additional file 1: Tables S2–S4).

## Discussion

An EPBVE ≥20%, a BMI ≥25 kg/m^2^, axillary clearance, re-excision, and infection were associated with low satisfaction regarding several aspects of the aesthetic result after conventional BCS. Regarding skin sensitivity in the breast, a BMI ≥25–<30 kg/m^2^, tumour size ≥15 mm, an EPBVE ≥20%, axillary clearance, re-excision, radiotherapy, and infection seemed to have had a negative impact.

### Aesthetic result

The great majority (84%) of patients were satisfied or very satisfied with the overall aesthetic result, which is in line with previous studies. Patient ratings as “excellent” or “good” were reported by Johansen et al. in 73%, by Taylor et al. in 87%, and by Cetintas et al. in 84%. Sneeuw et al. reported a rate of “very satisfied” in 59% and “little satisfied” in 30% [5, 9, 11, 21].

Age did not seem to influence patient satisfaction. This confirms results from some previous studies [5, 7, 9], whereas others have shown that both younger [13, 14] and older age [5, 11] can increase the risk of poor outcome. In these studies, age groups have been defined differently, but most studies have had a cutoff at 60 years to define “younger” and “older”. It could be hypothesized that a potential negative impact of high age on aesthetic outcome could be concealed by less favourable ratings from younger women, with possibly higher demands regarding the aesthetic result. In a study by Cetintas et al., age >50 years was shown to be a risk factor for poor aesthetic outcome when the evaluation of outcome was made by a panel of observers, but not when the patients evaluated the results [5].

Obesity (BMI ≥30 kg/m^2^) seemed to increase the risk of the patient not being satisfied with symmetry, as did re-excision. These results are in accordance with a study by Waljee et al. [13].

Tumour and breast size were not associated with patient satisfaction regarding aesthetic outcome in our study. Other studies have shown either no influence [3, 5, 9] or a negative influence [13] of larger tumour sizes on outcome. Most studies have shown a correlation between high specimen weight and worse aesthetic result [7, 11, 12]. However, the extent of excised tissue should be related to preoperative breast volume. Some studies have used bra cup size to estimate breast size [13], which is an imprecise measurement [22]. Bulstrode et al. [23] and Cochrane et al. [6] used mammography measurements, which improved accuracy. However, this method requires access to mammograms, applicable software, and calculation of breast volume. In our study, the estimation of breast volume was made using plastic cups, which are routinely used in our department. This is a valid method, with acceptable reliability, and is readily available in the clinical setting [17]. Our results indicate that an EPBVE exceeding 20% is a risk factor for poor outcome regarding several aspects of aesthetic outcome, which is in line with the studies mentioned above.

The location of the tumour did not seem to affect patient satisfaction. However, some subgroups consisted of very few patients, making it difficult to achieve statistical power. Some previous studies have shown a worse aesthetic outcome with tumours located medially or in the lower quadrants [12–14], while others have shown no difference [3–5, 7, 9].

In this study, axillary clearance appeared to be a strong determinant for poor satisfaction regarding overall aesthetic result, and remained statistically significant in the multivariable model. This contradicts studies showing no influence of the extent of axillary surgery on aesthetic outcome [3, 7, 11]. However, some previous studies have noted an effect of axillary surgery on the breast. Beadle et al. reported that while axillary clearance had not influenced overall aesthetic results, it did increase the risk of breast oedema up to over a year postoperatively [3]. In this study, there were unfortunately no reliable data concerning postoperative lymphedema, a condition which is likely to have had a negative impact on satisfaction. Another possible way for axillary clearance to impact the aesthetic outcome of the breast is if the axillary scar pulls the upper lateral quadrant up towards the axilla.

Radiotherapy seemed to negatively influence many aspects of the aesthetic outcome in our study, but the results were not statistically significant. However, some subgroups consisted of very few patients. Radiotherapy may impact the aesthetic outcome by influence on skin colour and changes in breast tissue, such as increased breast fibrosis. Several previous studies have shown a negative influence of radiotherapy on aesthetic results [3, 11, 12].

Chemotherapy and hormonal therapy were not associated with patient satisfaction in this study. Some previous studies have shown no influence of chemotherapy on aesthetic outcome [24], whereas others have shown that both sequential [8] and concomitant [11] chemotherapy may have a negative impact. Treatment with tamoxifen has not been shown to influence aesthetic results in previous studies, in accordance with our results [4, 5, 8, 11, 24, 25].

### Skin sensitivity

In regard to skin sensitivity in the operated breast, 67% were satisfied or very satisfied. In a study by Hau et al., 77% reported excellent/good/normal sensitivity vs. 23% fair/poor. These patients rated sensitivity 5 years postoperatively, which could explain the higher satisfaction rate. Hau et al. showed a correlation between impairment of breast sensitivity and worse quality of life (QoL) [26].

A BMI ≥25–<30 kg/m^2^, tumour size ≥15 mm, an EPBVE ≥20%, axillary clearance, re-excision, radiotherapy, and infection, seem to have negatively influenced patient satisfaction regarding skin sensitivity after conventional BCS in this study. Independent risk factors in the multivariable analysis were BMI, tumour size, re-excision, and infection. It is interesting that BMI ≥25–<30 kg/m^2^ seemed to be a risk factor, but not a BMI ≥30. An explanation for this could be that women with larger breasts may have experienced poor sensitivity in the breasts preoperatively. Previous studies have shown that breast reduction procedures can improve skin sensitivity in women with very large breasts [27].

To our knowledge, there are no previous studies investigating potential determinants influencing skin sensitivity after BCS. This may partly be caused by the fact that skin sensitivity in the breast is seldom studied exclusively, but is more often incorporated in an overall QoL score [9, 26].

Techniques of oncoplastic breast surgery are being more widely used. It could be hypothesized that more extensive mobilization or rearrangement of breast tissue could have a negative impact on skin sensitivity. This could be one reason for surgeons to advise against use of more advanced techniques. However, as we have shown, also in conventional BCS, patients can experience impaired skin sensitivity in the operated breast. Further studies to investigate this are needed in order to gain more knowledge, which could aid decision-making regarding which surgical technique to choose, and enhance the accuracy of information given to patients.

### Study strengths and limitations

The strengths of this study are the size of the study population and the prospective approach, with standardized measurements of tumour and anthropometric characteristics. Follow-up took place approximately 1 year after surgery and completion of radiotherapy, making it possible to study the effects of adjuvant therapy on outcome.

The main weakness of the study is that a fairly large proportion of the study population was lost in follow-up. This was partly caused by limited resources in the out-patient clinic during certain time periods, inhibiting invitations to follow-up. To investigate potential selection bias, participants and non-participants were compared (Additional file 1: Table S4). The characteristics are very similar, and we consider the material to be a representative study sample.

It has been difficult to define surgical techniques more precisely since several surgeons were involved, each deciding how much mobilization was needed in the individual patient. However, the aim of this study was not to compare surgical techniques, but to identify risk factors for an unsatisfying result after conventional BCS in general.

Complications, other than infection, may have influenced satisfaction in the individual patient. However, there were very few serious complications and it is unlikely that they would have had any significant impact on the results of the study population in general. Previous breast cancer surgery on the opposite breast could influence satisfaction with symmetry. A sensitivity analysis excluding 16 patients to which this applied was performed (Additional file 1: Table S5). The results were similar. However, in this analysis, a statistically significant association between adjuvant hormonal therapy and low satisfaction was observed. It could be hypothesized that a potential impact of hormonal therapy on patient weight and breast size would not cause as much asymmetry in patients who had undergone surgery and radiotherapy bilaterally, since the breasts would react similarly to weight fluctuations.

## Conclusions

This study supports previously published recommendations presenting a maximum limit of 20% breast volume excision with conventional BCS [22]. If the excision is estimated to exceed this, oncoplastic breast surgery or mastectomy with reconstruction might be more suitable surgical approaches to offer the patient.

## Additional file

Additional file 1: Supplemental material. p. 1: Calculation details of registration rate. p. 2: Fig S1. p. 3-4 Questionnaire. p. 5: Table S1. p. 6: Table S2. p. 7: Table S3. p. 8: Table S4. p. 9: Table S5. ﻿ (DOCX 247 kb)

## Abbreviations

BCS: Breast-conserving surgery
BMI: Body mass index
CI: Confidence interval
EPBVE: Estimated percentage of breast volume excised
INCA: Information Network for Cancer Care
IQR: Interquartile range
OR: Odds ratio
QoL: Quality of life
