# Supplementary material for: Determinants for patient satisfaction regarding aesthetic outcome and skin sensitivity after breast-conserving surgery
Source: World J Surg Oncol. 2016 Dec 7;14:303. doi: 10.1186/s12957-016-1053-8 (PMC5142134; doi:10.1186/s12957-016-1053-8)
Supplement: Additional file 1: — Supplemental material. p. 1: Calculation details of registration rate. p. 2: Fig S1. p. 3-4 Questionnaire. p. 5: Table S1. p. 6: Table S2. p. 7: Table S3. p. 8: Table S4. p. 9: Table S5. ﻿ (DOCX 247 kb) [file 12957_2016_1053_MOESM1_ESM.docx]

**Additional file 1**

**Supplemental material**

**Calculation details of registration rate**

The material was compared retrospectively to the Swedish Breast Cancer Registry (supplement fig. S1). In the same time period as inclusion in our study was proceeding, 748 breast cancer patients were reported in INCA as primarily operated with BCS at Skåne University Hospital Malmö, including those who had undergone a complementary mastectomy. When compared to our study sample, 16 women were not registered in INCA for unknown reasons. Thus, we considered the total number of women with breast cancer primarily operated in Malmö with BCS to be 764 (748+16).

Since INCA only includes breast cancer patients, 43 women in our study with a benign breast mass were withdrawn from the calculation of registration rate, as were those not operated (n=6) and those who underwent a primary mastectomy (n=24). In addition to the included participants, 19 women actively declined participation. Thus, our identified sample consisted of 599 patients (653-43-6-24+19). Hence, 78% (599/764) of potential participants were identified in the current study and were registered in the database. The number of patients not included for unknown reasons was 165 (764-599).

**Figure S1**

**Name**:…………………………………….

**Civil registration number**:…………

**Today’s date**:……………………….

**Questionnaire to patients operated due to breast cancer**

**or benign breast mass**

**– with or without operation of the other breast.**

It is optional to answer the questions in this questionnaire. The purpose of the questionnaire is to follow up and possibly enhance the quality of care given. The answers will be stored electronically and will be handled with the same level of confidentiality as other medical records. Your care will not be affected if you choose not to complete the questionnaire.

|  | **Not at all** | **Partly** | **Almost entirely** | **Entirely** |
| --- | --- | --- | --- | --- |
| **1.** Has the operation **met your**  **expectations** regarding the aesthetic result? |  |  |  |  |
| **2.** Did you receive **enough information** regarding the expected aesthetic result? |  |  |  |  |
| **3.** Did you feel that you could **take part in deciding** which operation should be done? |  |  |  |  |

**4.** **Has the entire breast been removed?**

Yes

No

-**if yes** continue to **question 8**

**5. If you were operated with breast-conserving surgery, how satisfied are you with:**

|  | **Dis-satisfied** | **Not entirely satisfied** | **Satisfied** | **Very satisfied** |
| --- | --- | --- | --- | --- |
| - a. the appearance of the breast after the operation performed about a year ago? |  |  |  |  |
| - b. the size of the cancer operated breast? |  |  |  |  |
| - c. the shape of the cancer operated breast? |  |  |  |  |
| - d. the location of the nipple/areola? |  |  |  |  |
| - e. the appearance of the nipple/areola? |  |  |  |  |
| - f. the similarity (symmetry) between the breasts? |  |  |  |  |
| - g. the appearance of the scars? |  |  |  |  |
| - h. skin sensitivity in the breast? |  |  |  |  |

Comments:………………………………………………………………………………………………………………………………………………………………………

Do not

know

Yes

No

**6.** Would you have preferred the entire breast **to be removed**?

**7.** Do you wish to have the cancer operated

No

Yes

breast **corrected**?

**If yes**, for what reason?..................................................................................

...................................................................................................................................

**8. Has your other breast been operated upon?**

**If yes**, please **answer the following question**:

**9. Has the entire other breast been removed?**

Yes No

**10. If the other breast has been operated upon, what was the reason?**

Cancer in the other breast Yes No

**If no, specify** the reason why the other breast was operated upon, for example size difference ………………………………………………………………………

……………………………………………………………………………………

**If the other breast was not entirely removed, please answer following questions:**

**11. How satisfied are you with the other breast´s**

|  | **Dis-satisfied** | **Not entirely satisfied** | **Satisfied** | **Very satisfied** |
| --- | --- | --- | --- | --- |
| - a. appearance? |  |  |  |  |
| - b. size? |  |  |  |  |
| - c. shape? |  |  |  |  |
| - d. the location of the nipple/areola? |  |  |  |  |
| - e. the appearance of the nipple/areola? |  |  |  |  |
| - f. the appearance of the scars? |  |  |  |  |
| - g. skin sensitivity in the breast? |  |  |  |  |

**12**. Do you wish to have further corrections of the other breast? Yes No

**13**. Are you aware that it is possible to **use an external prosthesis** to enhance the similarity of the breasts? Yes No

**If yes**, do you use a prosthesis?

Yes No

**If no**, would you like to try one? Yes No

Other comments:

…………………………………………………………………………………………………………………………………………………………………………………………………………………………………………………………………………

**Thank you for your participation!**

| **Table S1. Characteristics of participants and non-participants.** | | | |  |
| --- | --- | --- | --- | --- |
|  | Study population^a^ | Not complete^b^ | Not invited^c^ |  |
|  | n=297 | n=67 | n=123 |  |
| Age (years) | 62 (54-68; 34-85)^d^ | 55 (48-62)^e^ | 59 (49-67)^e^ |  |
| BMI (kg/m^2^) | 25 (23-29; 18-51)^d^ | 25.5 (22-30)^e^ | 26 (23-29)^e^ |  |
| Tumour size (mm) | 15 (10-20; 0,5-60)^d^ | 13.5 (9-20)^e^ | 15 (10-20)^e^ |  |
| Breast size (ml) | 500 (375-737.5; 160-1800)^d^ | 475 (360-790)^e^ | 510 (350-800)^e^ |  |
| EPBVE^f^ (%) | 12.4 (9.2-17.1; 1,3-73,8)^d^ | 11.3 (8.0-14.1)^e^ | 12.5 (9.4-16.4)^e^ |  |

a. Complete participants.

b. Patients who did not attend follow-up or complete questionnaire or questionnaire missing.

c. Patients who did not receive invitation to follow-up (administrative reasons).

d. Median (interquartile range; range)

e. Median (interquartile range)

f. Estimated percentage of Breast Volume Excised.

**Table S2. Satisfaction regarding shape of the operated breast.**

| Factor | Satisfied | Not satisfied | OR (95% CI) | OR (95% CI)^a^ | OR (95% CI)^b^ |
| --- | --- | --- | --- | --- | --- |
|  | n (%) | n (%) |  |  |  |
| Age (years) |  |  |  |  |  |
| <50 | 40 (16.9) | 6 (17.6) | 1 | 1 | 1 |
| ≥50-<65 | 100 (42.2) | 14 (41.2) | 0.93 (0.34-2.60) | 0.93 (0.33-2.61) | 0.89 (0.31-2.55) |
| ≥65 | 97 (40.9) | 14 (41.2) | 0.96 (0.35-2.68) | 0.84 (0.30-2.40) | 0.77 (0.27-2.25) |
|  |  |  |  |  |  |
| BMI (kg/m^2^) | |  |  |  |  |
| <25 | 109 (46.2) | 10 (29.4) | 1 | 1 | 1 |
| ≥25-<30 | 75 (31.8) | 16 (47.1) | 2.33 (1.00-5.40) | 2.37 (1.01-5.55) | 2.47 (1.03-5.88) |
| ≥30 | 52 (22.0) | 8 (23.5) | 1.68 (0.63-4.50) | 1.72 (0.63-4.67) | 2.08 (0.74-5.82) |
| Missing | 1 |  |  |  |  |
|  |  |  |  |  |  |
| Breast volume (ml)^c^ | |  |  |  |  |
| <500 | 106 (45.1) | 14 (41.2) | 1 | 1 |  |
| ≥500 | 129 (54.9) | 20 (58.8) | 1.17 (0.57-2.44) | 1.00 (0.43-2.32) |  |
| Missing | 2 |  |  |  |  |
|  |  |  |  |  |  |
| Tumour size (mm)^c^ | |  |  |  |  |
| <15 | 113 (47.7) | 12 (35.3) | 1 | 1 |  |
| ≥15 | 124 (52.3) | 22 (64.7) | 1.67 (0.79-3.53) | 1.56 (0.73-3.35) |  |
|  |  |  |  |  |  |
| Specimen weight (g)^c^ | |  |  |  |  |
| <63 | 121 (51.1) | 13 (38.2) | 1 | 1 |  |
| ≥63 | 116 (48.9) | 21 (61.8) | 1.69 (0.81-3.52) | 1.54 (0.71-3.35) |  |
|  |  |  |  |  |  |
| EPBVE^d^ (%) | |  |  |  |  |
| <10 | 75 (31.9) | 6 (17.6) | 1 | 1 | 1 |
| ≥10-<20 | 131 (55.7) | 18 (52.9) | 1.72 (0.65-4.52) | 1.87 (0.70-5.00) | 1.87 (0.70-5.00) |
| ≥20 | 29 (12.3) | 10 (29.4) | 4.31 (1.44-12.94) | 4.79 (1.55-14.77) | 4.79 (1.55-14.77) |
| Missing | 2 |  |  |  |  |
|  |  |  |  |  |  |
| Axillary clearance | |  |  |  |  |
| No | 198 (83.9) | 25(73.5) | 1 | 1 |  |
| Yes | 38 (16.1) | 9 (26.5) | 1.88 (0.81-4.33) | 1.65 (0.70-3.90) |  |
| Missing | 1 |  |  |  |  |
|  |  |  |  |  |  |
| Re-excision |  |  |  |  |  |
| No | 220 (92.8) | 31 (91.2) | 1 | 1 |  |
| Yes | 17 (7.2) | 3 (8.8) | 1.25 (0.35-4.52) | 1.38 (0.37-5.12) |  |
|  |  |  |  |  |  |
| Infection |  |  |  |  |  |
| No | 222 (93.7) | 29 (85.3) | 1 | 1 |  |
| Yes | 15 (6.3) | 5 (14.7) | 2.55 (0.86-7.54) | 2.70 (0.88-8.30) |  |
|  |  |  |  |  |  |
| Radiotherapy | |  |  |  |  |
| No | 31 (13.1) | 2 (5.9) | 1 | 1 |  |
| Yes | 206 (86.9) | 32 (94.1) | 2.41 (0.55-10.55) | 2.24 (0.50-10.11) |  |
|  |  |  |  |  |  |
| Chemotherapy | |  |  |  |  |
| No | 208 (87.8) | 31 (91.2) | 1 | 1 |  |
| Yes | 29 (12.2) | 3 (8.8) | 0.69 (0.20-2.42) | 0.60 (0.16-2.21) |  |
|  |  |  |  |  |  |
| Hormonal therapy | |  |  |  |  |
| No | 107 (45.1) | 11 (32.4) | 1 | 1 |  |
| Yes | 130 (54.9) | 23 (67.6) | 1.72 (0.80-3.69) | 1.63 (0.75-3.54) |  |
|  |  |  |  |  |  |
| Quadrant^e^ |  |  |  |  |  |
| UOQ | 122 (51.7) | 18 (52.9) | 1 | 1 |  |
| LOQ | 48 (20.3) | 4 (11.8) | 0.57 (0.18-1.76) | 0.53 (0.17-1.67) |  |
| LIQ | 17 (7.2) | 6 (17.6) | 2.39 (0.83-6.86) | 2.48 (0.84-7.33) |  |
| UIQ | 47 (19.9) | 6 (17.6) | 0.87 (0.32-2.31) | 0.79 (0.29-2.14) |  |
| Central | 2 (0.8) | 0 (0.0) | --- | --- |  |
| Missing |  | 1 |  |  |  |

a. Adjusted for age and BMI. b. Adjusted for age, BMI and EPBVE. c. Groups divided at the median. d. Estimated Percentage of Breast Volume Excised. e. UOQ=upper outer quadrant; LOQ=lower outer quadrant; LIQ=lower inner quadrant; UIQ=upper inner quadrant

**Table S3. Satisfaction regarding size of the operated breast.**

| Factor | Satisfied | Not satisfied | OR (95% CI) | OR (95% CI)^a^ | OR (95% CI)^b^ |
| --- | --- | --- | --- | --- | --- |
|  | n (%) | n (%) |  |  |  |
| Age (years) |  |  |  |  |  |
| <50 | 39 (16.5) | 7 (18.4) | 1 | 1 | 1 |
| ≥50-<65 | 96 (40.7) | 18 (47.4) | 1.05 (0.40-2.70) | 0.99 (0.38-2.59) | 1.00 (0.37-2.69) |
| ≥65 | 101 (42.8) | 13 (34.2) | 0.72 (0.27-1.93) | 0.61 (0.22-1.66) | 0.59 (0.21-1.65) |
|  |  |  |  |  |  |
| BMI (kg/m^2^) | |  |  |  |  |
| <25 | 107 (45.5) | 13 (34.2) | 1 | 1 | 1 |
| ≥25-<30 | 80 (34.0) | 12 (31.6) | 1.24 (0.54-2.85) | 1.33 (0.57-3.11) | 1.19 (0.49-2.84) |
| ≥30 | 48 (20.4) | 13 (34.2) | 2.23 (0.96-5.17) | 2.48 (1.05-5.85) | 2.67 (1.09-6.52) |
| Missing | 1 |  |  |  |  |
|  |  |  |  |  |  |
| Breast volume (ml)^c^ | |  |  |  |  |
| <500 | 104 (44.4) | 15 (39.5) | 1 | 1 |  |
| ≥500 | 130 (55.6) | 23 (60.5) | 1.23 (0.61-2.47) | 0.94 (0.41-2.18) |  |
| Missing | 2 |  |  |  |  |
|  |  |  |  |  |  |
| Tumour size (mm)^c^ | |  |  |  |  |
| <15 | 114 (48.3) | 13 (34.2) | 1 | 1 |  |
| ≥15 | 122 (51.7) | 25 (65.8) | 1.78 (0.88-3.68) | 1.82 (0.87-3.79) |  |
|  |  |  |  |  |  |
| Specimen weight (g)^c^ | |  |  |  |  |
| <63 | 119 (50.4) | 15 (39.5) | 1 | 1 |  |
| ≥63 | 117 (49.6) | 23 (60.5) | 1.56 (0.78-3.14) | 1.36 (0.65-2.87) |  |
|  |  |  |  |  |  |
| EPBVE^d^ (%) | |  |  |  |  |
| <10 | 74 (31.6) | 9 (23.7) | 1 | 1 | 1 |
| ≥10-<20 | 130 (55.6) | 20 (52.6) | 1.27 (0.55-2.92) | 1.41 (0.60-3.35) | 1.39 (0.58-3.33) |
| ≥20 | 30 (12.8) | 9 (23.7) | 2.47 (0.89-6.82) | 3.09 (1.08-8.85) | 2.87 (0.99-8.35) |
| Missing | 2 |  |  |  |  |
|  |  |  |  |  |  |
| Axillary clearance | |  |  |  |  |
| No | 199 (84.7) | 27 (71.1) | 1 | 1 | 1 |
| Yes | 36 (15.3) | 11 (28.9) | 2.25 (1.03-4.94) | 2.23 (0.99-5.01) | 2.12 (0.93-4.81) |
| Missing | 1 |  |  |  |  |
|  |  |  |  |  |  |
| Re-excision |  |  |  |  |  |
| No | 218 (92.4) | 36 (94.7) | 1 | 1 |  |
| Yes | 18 (7.6) | 2 (5.3) | 0.67 (0.15-3.02) | 0.81 (0.18-3.71) |  |
|  |  |  |  |  |  |
| Infection |  |  |  |  |  |
| No | 222 (94.1) | 33 (86.8) | 1 | 1 |  |
| Yes | 14 (5.9) | 5 (13.2) | 2.40 (0.81-7.11) | 2.02 (0.66-6.19) |  |
|  |  |  |  |  |  |
| Radiotherapy | |  |  |  |  |
| No | 28 (11.9) | 5 (13.2) | 1 | 1 |  |
| Yes | 208 (88.1) | 33 (86.8) | 0.89 (0.32-2.46) | 0.88 (0.30-2.55) |  |
|  |  |  |  |  |  |
| Chemotherapy | |  |  |  |  |
| No | 207 (87.7) | 34 (89.5) | 1 | 1 |  |
| Yes | 29 (12.3) | 4 (10.5) | 0.84 (0.28-2.54) | 0.75 (0.24-2.40) |  |
|  |  |  |  |  |  |
| Hormonal therapy | |  |  |  |  |
| No | 108 (45.8) | 13 (34.2) | 1 | 1 |  |
| Yes | 128 (54.2) | 25 (65.8) | 1.62 (0.79-3.33) | 1.69 (0.81-3.53) |  |
|  |  |  |  |  |  |
| Quadrant^e^ |  |  |  |  |  |
| UOQ | 120 (51.1) | 22 (57.9) | 1 | 1 |  |
| LOQ | 46 (19.6) | 6 (15.8) | 0.71 (0.27-1.87) | 0.68 (0.25-1.81) |  |
| LIQ | 19 (8.1) | 4 (10.5) | 1.15 (0.36-3.70) | 1.28 (0.39-4.24) |  |
| UIQ | 48 (20.4) | 6 (15.8) | 0.68 (0.26-1.79) | 0.68 (0.26-1.80) |  |
| Central | 2 (0.9) | 0 (0.0) | --- | --- |  |
| Missing |  | 1 |  |  |  |

a. Adjusted for age and BMI. b. Adjusted for age, BMI, EPBVE and axillary clearance. c. Groups divided at the median. d. Estimated Percentage of Breast Volume Excised. e. UOQ=upper outer quadrant; LOQ=lower outer quadrant; LIQ=lower inner quadrant; UIQ=upper inner quadrant

**Table S4. Satisfaction regarding visual appearance of the scar.**

| Factor | Satisfied | Not satisfied | OR (95% CI) | OR (95% CI)^a^ | OR (95% CI)^b^ |
| --- | --- | --- | --- | --- | --- |
|  | n (%) | n (%) |  |  |  |
| Age (years) |  |  |  |  |  |
| <50 | 44 (18.3) | 2 (6.1) | 1 | 1 | 1 |
| ≥50-<65 | 99 (41.3) | 16 (48.5) | 3.56 (0.78-16.13) | 3.56 (0.78-16.18) | 3.53 (0.77-16.24) |
| ≥65 | 97 (40.4) | 15 (45.5) | 3.40 (0.75-15.52) | 3.32 (0.72-15.29) | 3.24 (0.70-15.09) |
|  |  |  |  |  |  |
| BMI (kg/m^2^) | |  |  |  |  |
| <25 | 107 (44.8) | 13 (39.4) | 1 | 1 | 1 |
| ≥25-<30 | 78 (32.6) | 14 (42.4) | 1.48 (0.66-3.32) | 1.46 (0.64-3.34) | 1.48 (0.64-3.42) |
| ≥30 | 54 (22.6) | 6 (18.2) | 0.92 (0.33-2.54) | 0.87 (0.31-2.46) | 0.95 (0.33-2.74) |
| Missing | 1 |  |  |  |  |
|  |  |  |  |  |  |
| Breast volume (ml)^c^ | |  |  |  |  |
| <500 | 102 (43.0) | 17 (51.5) | 1 | 1 |  |
| ≥500 | 135 (57.0) | 16 (48.5) | 0.71 (0.34-1.48) | 0.64 (0.27-1.51) |  |
| Missing | 3 |  |  |  |  |
|  |  |  |  |  |  |
| Tumour size (mm)^c^ | |  |  |  |  |
| <15 | 117 (48.8) | 12 (36.4) | 1 | 1 |  |
| ≥15 | 123 (51.2) | 21 (63.6) | 1.67 (0.78-3.54) | 1.57 (0.73-3.39) |  |
|  |  |  |  |  |  |
| Specimen weight (g)^c^ | |  |  |  |  |
| <63 | 123 (51.2) | 13 (39.4) | 1 | 1 |  |
| ≥63 | 117 (48.8) | 20 (60.6) | 1.62 (0.77-3.40) | 1.69 (0.77-3.71) |  |
|  |  |  |  |  |  |
| EPBVE^d^ (%) | |  |  |  |  |
| <10 | 76 (32.1) | 7 (21.2) | 1 | 1 | 1 |
| ≥10-<20 | 132 (55.7) | 17 (51.5) | 1.40 (0.56-3.52) | 1.35 (0.53-3.46) | 1.35 (0.53-3.46) |
| ≥20 | 29 (12.2) | 9 (27.3) | 3.37 (1.15-9.89) | 3.19 (1.07-9.51) | 3.19 (1.07-9.51) |
| Missing | 3 |  |  |  |  |
|  | |  |  |  |  |
| Axillary clearance | |  |  |  |  |
| No | 198 (82.8) | 27 (81.8) | 1 | 1 |  |
| Yes | 41 (17.2) | 6 (18.2) | 1.07 (0.42-2.77) | 1.08 (0.41-2.82) |  |
| Missing | 1 |  |  |  |  |
|  |  |  |  |  |  |
| Re-excision |  |  |  |  |  |
| No | 224 (93.3) | 30 (90.9) | 1 | 1 |  |
| Yes | 16 (6.7) | 3 (9.1) | 1.40 (0.39-5.09) | 1.32 (0.36-4.92) |  |
|  |  |  |  |  |  |
| Infection |  |  |  |  |  |
| No | 226 (94.2) | 28 (84.8) | 1 | 1 |  |
| Yes | 14 (5.8) | 5 (15.2) | 2.88 (0.97-8.61) | 3.15 (1.00-9.89) |  |
|  |  |  |  |  |  |
| Radiotherapy | |  |  |  |  |
| No | 33 (13.8) | 3 (9.1) | 1 | 1 |  |
| Yes | 207 (86.3) | 30 (90.9) | 1.59 (0.46-5.52) | 1.30 (0.36-4.65) |  |
|  |  |  |  |  |  |
| Chemotherapy | |  |  |  |  |
| No | 212 (88.3) | 29 (87.9) | 1 | 1 |  |
| Yes | 28 (11.7) | 4 (12.1) | 1.04 (0.34-3.19) | 1.40 (0.43-4.52) |  |
|  |  |  |  |  |  |
| Hormonal therapy | |  |  |  |  |
| No | 107 (44.6) | 15 (45.5) | 1 | 1 |  |
| Yes | 133 (55.4) | 18 (54.5) | 0.97 (0.47-2.01) | 0.97 (0.46-2.03) |  |
|  |  |  |  |  |  |
| Quadrant^e^ |  |  |  |  |  |
| UOQ | 125 (52.3) | 16 (48.5) | 1 | 1 |  |
| LOQ | 47 (19.7) | 6 (18.2) | 1.00 (0.37-2.70) | 0.98 (0.36-2.68) |  |
| LIQ | 19 (7.9) | 4 (12.1) | 1.65 (0.50-5.45) | 1.50 (0.45-5.05) |  |
| UIQ | 46 (19.2) | 7 (21.2) | 1.19 (0.46-3.08) | 1.08 (0.41-2.85) |  |
| Central | 2 (0.8) | 0 (0.0) | --- | --- |  |
| Missing | 1 |  |  |  |  |

a. Adjusted for age and BMI. b. Adjusted for age, BMI, and EPBVE. c. Groups divided at the median. d. Estimated Percentage of Breast Volume Excised. e. UOQ=upper outer quadrant; LOQ=lower outer quadrant; LIQ=lower inner quadrant; UIQ=upper inner quadrant

**Table S5. Satisfaction with symmetry.** (Patients with op. opposite breast excluded)

| Factor | Satisfied | Not satisfied | OR (95% CI) | OR (95% CI)^a^ | OR (95% CI)^b^ |
| --- | --- | --- | --- | --- | --- |
|  | n (%) | n (%) |  |  |  |
| Age (years) |  |  |  |  |  |
| <50 | 35 (17.9) | 10 (18.5) | 1 | 1 | 1 |
| ≥50-<65 | 79 (40.5) | 24 (44.4) | 1.06 (0.46-2.46) | 1.03 (0.44-2.43) | 1.05 (0.43-2.60) |
| ≥65 | 81 (41.5) | 20 (37.0) | 0.86 (0.37-2.04) | 0.71 (0.29-1.71) | 0.66 (0.26-1.67) |
|  |  |  |  |  |  |
| BMI (kg/m^2^) | |  |  |  |  |
| <25 | 92 (47.4) | 17 (31.5) | 1 | 1 | 1 |
| ≥25-<30 | 63 (32.5) | 19 (35.2) | 1.63 (0.79-3.38) | 1.75 (0.83-3.67) | 1.66 (0.75-3.67) |
| ≥30 | 39 (20.1) | 18 (33.3) | 2.50 (1.17-5.35) | 2.73 (1.25-5.96) | 2.54 (1.04-6.23) |
| Missing | 1 |  |  |  |  |
|  |  |  |  |  |  |
| Breast volume (ml)^c^ | |  |  |  |  |
| <500 | 91 (47.2) | 19 (35.2) | 1 | 1 |  |
| ≥500 | 102 (52.8) | 35 (64.8) | 1.64 (0.88-3.07) | 1.30 (0.63-2.70) |  |
| Missing | 2 |  |  |  |  |
|  |  |  |  |  |  |
| Tumour size (mm)^c^ | |  |  |  |  |
| <15 | 92 (47.2) | 20 (37.0) | 1 | 1 |  |
| ≥15 | 103 (52.8) | 34 (63.0) | 1.52 (0.82-2.82) | 1.49 (0.79-2.83) |  |
|  |  |  |  |  |  |
| Specimen weight (g)^c^ | |  |  |  |  |
| <65 | 107 (54.9) | 16 (29.6) | 1 | 1 | 1 |
| ≥65 | 88 (45.1) | 38 (70.4) | 2.89 (1.51-5.52) | 2.62 (1.33-5.17) | 2.08 (1.02-4.26) |
|  |  |  |  |  |  |
| EPBVE^d^ (%) | |  |  |  |  |
| <10 | 65 (33.7) | 14 (25.9) | 1 | 1 |  |
| ≥10-<20 | 105 (54.4) | 30 (55.6) | 1.33 (0.66-2.69) | 1.56 (0.75-3.24) |  |
| ≥20 | 23 (11.9) | 10 (18.5) | 2.02 (0.79-5.17) | 2.56 (0.96-6.83) |  |
| Missing | 2 |  |  |  |  |
|  |  |  |  |  |  |
| Axillary clearance | |  |  |  |  |
| No | 162 (83.5) | 42 (77.8) | 1 | 1 |  |
| Yes | 32 (16.5) | 12 (22.2) | 1.45 (0.69-3.05) | 1.33 (0.61-2.87) |  |
| Missing | 1 |  |  |  |  |
|  |  |  |  |  |  |
| Re-excision |  |  |  |  |  |
| No | 185 (94.9) | 45 (83.3) | 1 | 1 | 1 |
| Yes | 10 (5.1) | 9 (16.7) | 3.70 (1.42-9.64) | 4.93 (1.81-13.37) | 4.47 (1.52-13.14) |
|  |  |  |  |  |  |
| Infection |  |  |  |  |  |
| No | 185 (94.9) | 46 (85.2) | 1 | 1 | 1 |
| Yes | 11 (5.1) | 8 (14.8) | 3.22 (1.20-8.61) | 2.70 (0.98-7.47) | 2.30 (0.80-6.59) |
|  |  |  |  |  |  |
| Radiotherapy | |  |  |  |  |
| No | 27 (13.8) | 4 (7.4) | 1 | 1 |  |
| Yes | 168 (86.2) | 50 (92.6) | 2.01 (0.67-6.01) | 2.10 (0.67-6.53) |  |
|  |  |  |  |  |  |
| Chemotherapy | |  |  |  |  |
| No | 172 (88.2) | 48 (88.9) | 1 | 1 |  |
| Yes | 23 (11.8) | 6 (11.1) | 0.94 (0.36-2.43) | 0.82 (0.30-2.27) |  |
|  |  |  |  |  |  |
| Hormonal therapy | |  |  |  |  |
| No | 91 (46.7) | 17 (31.5) | 1 | 1 | 1 |
| Yes | 104 (53.3) | 37 (68.5) | 1.90 (1.01-3.61) | 1.95 (1.01-3.76) | 2.07 (1.03-4.16) |
|  |  |  |  |  |  |
| Quadrant^e^ |  |  |  |  |  |
| UOQ | 97 (49.7) | 31 (58.5) | 1 | 1 |  |
| LOQ | 39 (20.0) | 9 (17.0) | 0.72 (0.32-1.66) | 0.68 (0.29-1.58) |  |
| LIQ | 16 (8.2) | 4 (7.5) | 0.78 (0.24-2.52) | 0.80 (0.24-2.64) |  |
| UIQ | 41 (21.0) | 9 (17.0) | 0.69 (0.30-1.57) | 0.68 (0.29-1.56) |  |
| Central | 2 (1.0) | 0 (0.0) | --- | --- |  |
| Missing |  | 1 |  |  |  |

a. Adjusted for age and BMI. b. Adjusted for age, BMI, specimen weight, re-excision, infection and hormonal therapy. c. Groups divided at the median. d. Estimated Percentage of Breast Volume Excised. e. UOQ=upper outer quadrant; LOQ=lower outer quadrant; LIQ=lower inner quadrant; UIQ=upper inner quadrant
